# Immune responses in diabetic nephropathy: Pathogenic mechanisms and therapeutic target

**DOI:** 10.3389/fimmu.2022.958790

**Published:** 2022-08-15

**Authors:** Jiahao Chen, Qinhui Liu, Jinhan He, Yanping Li

**Affiliations:** Department of Pharmacy, Institute of Metabolic Diseases and Pharmacotherapy, West China Hospital, Sichuan University, Chengdu, China

**Keywords:** diabetic nephropathy, immune responses, therapeutic target, inflammation, pathogenesis

## Abstract

Diabetic nephropathy (DN) is a chronic, inflammatory disease affecting millions of diabetic patients worldwide. DN is associated with proteinuria and progressive slowing of glomerular filtration, which often leads to end-stage kidney diseases. Due to the complexity of this metabolic disorder and lack of clarity about its pathogenesis, it is often more difficult to diagnose and treat than other kidney diseases. Recent studies have highlighted that the immune system can inadvertently contribute to DN pathogenesis. Cells involved in innate and adaptive immune responses can target the kidney due to increased expression of immune-related localization factors. Immune cells then activate a pro-inflammatory response involving the release of autocrine and paracrine factors, which further amplify inflammation and damage the kidney. Consequently, strategies to treat DN by targeting the immune responses are currently under study. In light of the steady rise in DN incidence, this timely review summarizes the latest findings about the role of the immune system in the pathogenesis of DN and discusses promising preclinical and clinical therapies.

## Introduction

Diabetic nephropathy (DN) occurs in 20-50% of patients with diabetes and is the major risk for end-stage kidney disease (ESKD) (1). In 2019, 2.6 million new cases of DN were reported worldwide, and this incidence is predicted to increase in the future (2). Given the financial burden and lower quality of life associated with DN, understanding its molecular causes is of important for effective intervention and prevention.

DN is a clinical syndrome characterized by persistent albuminuria and a progressive decline in renal function, and it presents a typical pattern of glomerular disease (3). DN involves both changes in renal structure and function (4). Structurally, DN pathological features consist of glomerular mesangial expansion, basement membrane thickening, podocytes loss, nodular glomerulosclerosis and endothelial cells destruction (5). In the early stage of DN, there is tubular hypertrophy, but it is eventually processes to interstitial fibrosis with tubular atrophy. In the advanced stage, the injured kidney is infiltrated by immune cells (6). Functionally, DN shows increased albumin excretion and impaired glomerular filtration rate (7).

Historically, DN has not been considered an immune-mediated disease, but rather a disorder mediated by metabolic and hemodynamic factors (8). The progression of DN is highly unpredictable and it often occurs slowly over many years. In many countries, renal biopsy is rarely performed in patients with diabetes. It is only investigated when there is a significant increase in albuminuria or substantial decrease in renal function, which allow physicians to determine whether there is another kidney problem or comorbidities (1, 2, 9). Consequently, renal biopsies are usually performed in advanced stages of DN. This has severely hindered researchers to elucidate the role of immune system in progression of DN. Nevertheless, studies have been able to uncover a central role for immune-mediated inflammation in DN, involving both the innate and adaptive branches (7, 8, 10–12). Macrophages, as the predominant innate immune cells in DN, are commonly observed in the glomeruli and interstitium in experimental DN models and clinical trials at all stages of DN (13–15). The adaptive immune system mainly comprises T cells and B cells. The progression of DN correlates with activation of T cells in the blood and elevated numbers of CD4^+^ T cells in the kidney (11, 16, 17).

**Figure 1:**
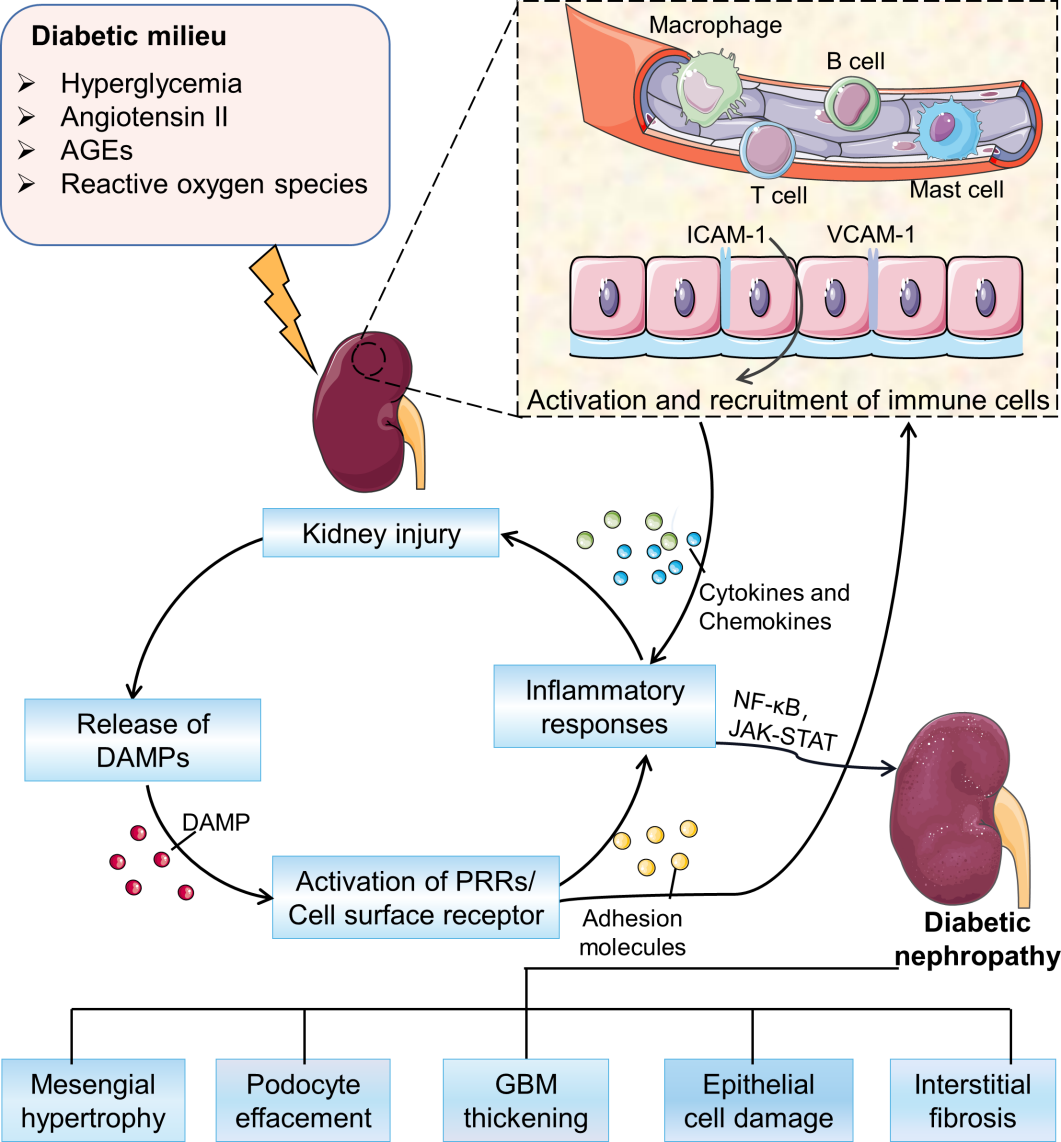
Overview of the pathogenesis of DN. In the diabetic milieu, hyperglycemia, advanced glycation end-products (AGEs), angiotensin II, and oxidative stress activate a variety of signaling cascades driving the recruitment and activation of immune cells to promote the development of inflammation and ultimately leading to a series of pathological changes in DN. AGEs, advanced glycation end products; DAMPs, damage associated molecular patterns; PRRs, pattern recognition receptors; GBM, glomerular basement membrane.

The immune pathogenic mechanism of DN is complex and involves the interaction of multiple pathways (**Figure 1**). In a diabetic mellitus, hyperglycemia and high lipid levels, including oxidative stress, reactive oxygen species (ROS), and oxidized lipids, damage kidney cells, leading to the release of damage-associated molecular patterns (DMAPs), and then trigger the pro-inflammatory signaling pathways (18). Besides, glycated proteins, such as advanced glycation end products (AGEs), can directly activate the complement system and trigger pro-inflammatory signaling (19). In response to continuous activation of innate immune injury, renal mesangial cells, endothelial cells and podocytes produce a variety of inflammatory mediators, including cytokines, chemokines, and adhesion molecules. These activate and recruit monocytes and macrophages, leading to further inflammatory cascade responses (7). The sustained chronic inflammation eventually drives the remodeling of renal structure and tubulointerstitial fibrosis (20–23).

Various subsets of kidney cells in DN overexpress cell adhesion molecules, which are proteins on the cell surface to bind or attach immune cells to ECM. These cell adhesion molecules recruit immune cells to the kidney (24). The immune cells express transcription factors as well as secrete cytokines and chemokines that work together to induce a pro-inflammatory response to exacerbate disease pathology (4). These insights of the involvement of the immune system in DN may lead to more effective treatments than the current strategies of blood glucose control and inhibition of the renin-angiotensin system. In this review, we provide an overview of the contribution by the immune system to DN pathogenesis, and we explore current efforts to treat the disease by targeting immune-related factors.

## Immune cells involved in DN pathogenesis

### Macrophages

**Figure 2:**
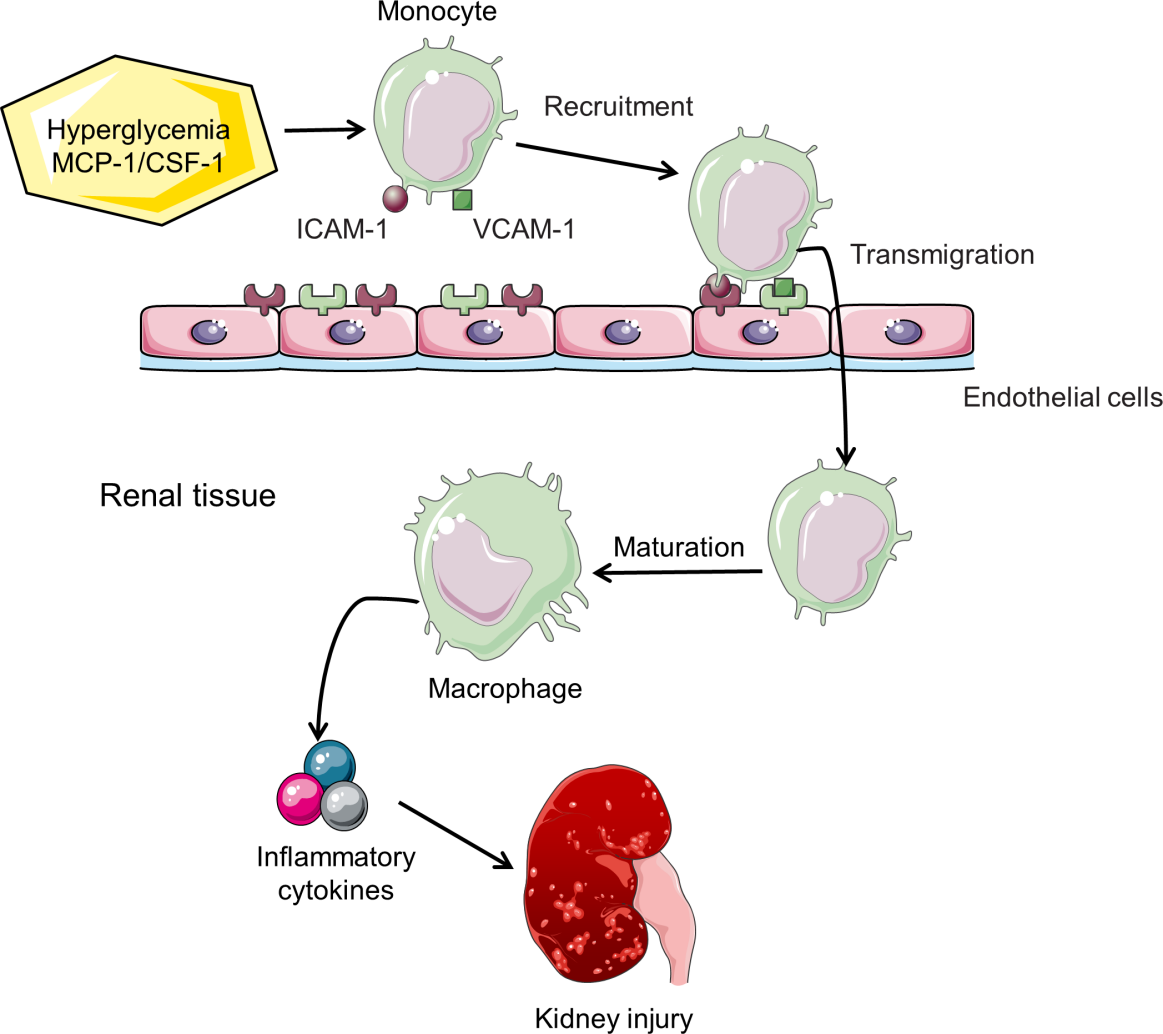
Macrophage recruitment and activation in DN. Hyperglycemia induces increased expression of cell adhesion molecules (ICAM-1/VCAM-1) and chemokines (MCP-1/CSF-1), thereby enhancing the recruitment of monocytes to the kidney. Chemokines also promote transendothelial migration. Monocytes mature into macrophages and subsequently release inflammatory cytokines, leading to the progression of DN.

Macrophages are the most important type of infiltrating immune cells in renal biopsies from experimental animal models and clinical patients with DN (25). The accumulation of F4/80- or CD68-positive macrophages detected by immunohistochemical staining or flow cytometry has been a characteristic feature of DN (26, 27). In mice with type 1 or 2 diabetes, macrophages accumulate in kidneys and become activated, which is associated with persistent hyperglycemia, deposition of glomerular immune complex, and increased production of chemokine, ultimately leading to renal injury and fibrosis (14, 15). Although detailed molecular mechanisms of macrophage migration and homing to the kidney have not been fully elucidated, cell adhesion molecules and chemokines/chemokine receptors are involved in this process. The vascular endothelium overexpresses cell adhesion molecules in its surface, such as intercellular adhesion molecule-1 (ICAM-1) and vascular cell adhesion molecule-1 (VCAM-1), which capture circulating macrophage precursors (28, 29). Mesangial cells, podocytes, and tubular epithelial cells are stimulated to secrete monocyte chemoattractant protein-1 (MCP-1) and osteopontin to facilitate migration of macrophages across the vascular endothelium and within the kidney (30–33). Renal parenchymal cells in diabetic mice also produce macrophage colony stimulating factor 1 (CSF-1), which promotes proliferation of kidney macrophages (**Figure 2**) (15, 25, 34).

Several factors promote the homing of macrophages to the kidney in the diabetic environment. Hyperglycemia and AGEs stimulate renal tubular cells expressing ICAM-1 and MCP-1 in the diabetic milieu, which promotes the recruitment of macrophages (32, 35). Once macrophages recruit to the diabetic kidney, local high glucose levels, AGEs and oxidized low-density lipoprotein (Ox-LDL) stimulate macrophages to release inflammatory cytokines (24). Other factors by which macrophages promote DN progression include production of ROS and proteases (24). These processes will aggravate tissue injury and ultimately lead to renal fibrosis.

Macrophages are plastic, pluripotent cells whose functions can change dramatically according to the microenvironment. Macrophages are classified as being “classically activated” (type M1) or “alternatively activated” (type M2) (36, 37). M1 macrophages perform immune surveillance function by secreting pro-inflammatory cytokines and chemokines and presenting antigen on their surface in order to stimulate other immune cells. M2 macrophages play an important role in immune regulation by secreting inhibitory cytokines and down-regulating immune response; they are inefficient at presenting antigens (38). Macrophages at sites of diabetic kidney injury are mainly of the M1 type (14, 15, 39).

Studies have shown that increased numbers of M1 macrophages are associated with severe DN lesions in mice lacking cyclooxygenase-2 (COX-2), an enzyme involved in metabolic processes preceding inflammation (40). The “triggering receptor expressed on myeloid cells”-1 (TREM-1) is an activating receptor of the immunoglobulin superfamily present on human myeloid cells. It can polarize macrophages toward the M2 type, thus reducing renal inflammation *in vitro* and *in vivo* (41). Mesenchymal stem cells (MSCs) also polarize macrophages towards the M2 phenotype and prevent renal injury in mouse models of DN. Interestingly, these effects are abolished in DN mouse models that have been treated with clodronate liposomes to deplete macrophages, suggesting that M2-type macrophages are necessary for renal protection. The ability of MSCs to polarize macrophages towards M2 appears to involve the activity of transcription factor EB (TFEB), which restores intracellular lysosomal function and autophagy activity, helping MSCs suppress the inflammatory response and alleviate renal injuries (42).

### T cells

T cells, which recruit to the diabetic kidney accompanying by the recruitment of macrophages, also contributes to the progression of DN. Although several previous studies have shown that the number of CD4^+^ T cells in renal interstitium correlates with the albuminuria level in DN animal models (17, 43, 44), the mechanism by which T cells home to the kidney in diabetes is poorly understood. Adhesion molecules and chemokines are reported to be involved in T cell recruitment (24). Leukocyte function-associated antigen 1 (LFA-1), which is expressed on T cells, could combine with ICAM-1 expressed on renal endothelial cells, tubular epithelial cells, and mesangial cells to promote T cell migration to kidney (45). CD4^+^ T cells were increased in the glomeruli of *db/db* diabetic mice, but this increase was abolished in the kidneys of ICAM-1 knockout *db/db* mice (35), suggesting that the interaction of LFA-1 with ICAM-1 plays a significant role in the recruitment of T cells to kidney. Activated T cells will secrete inflammatory cytokines such as interferon gamma (IFN-γ) and TNF-α (17). These inflammatory cytokines directly damage the kidney through cytotoxic effects and indirectly promote the homing and activation of macrophages (16). In addition, AGEs can bind to the AGE receptor expressed on T cells, which in turn stimulates T cells to secrete IFN-γ, leading to kidney inflammation (46).

T cells can be divided into many subsets according to their function and specific markers. Flow cytometry, immunohistochemistry, and immunofluorescence staining techniques are generally used to distinguish different T cell subtypes (11, 39). It is well-known that CD4^+^ T cells can differentiate into T-helper (Th) 1 cells, Th2 cells, Th17 cells, and Treg cells, which mainly produce IFN-γ, interleukin (IL)-4, IL-17 and Foxp3, respectively (47, 48). As the many subsets of T cells indicates, their roles are varied when the adaptive immune response is activated in DN pathogenesis (49, 50). The Th1 cell response precedes and accompanies type 1 diabetes (51). Increased levels of ICAM-1, P-selectin, IFN-γ and migration inhibitory factor in the kidney of mice with diabetes mellitus are associated with the homing of effector Th1 cells to the glomerulus (16, 52). Similarly, T-helper 17 cells secret IL-17 to elicit a strong pro-inflammatory response (53). Neutralization of IL-17A blocks NF-kB activation and the subsequent upregulation of proinflammatory genes, which in turn inhibits infiltration of the kidney by inflammatory cells (54). In contrast, Th2 cells produce IL-4 to promote humoral immunity, inhibit Th1 activation, and inhibit inflammation and fibrosis, providing an overall immunosuppressive effect (55). Furthermore, transfer of CD4^+^-Foxp3^+^ Treg cells improves insulin resistance and ameliorates DN pathogenesis in mice by tipping the balance toward anti-inflammation and suppressing CD8^+^ T cells infiltration in the kidneys and adipose tissue (47, 56, 57). CD8^+^ T cells are predominantly cytotoxic and damage the kidney by direct cell-cell signaling *via* surface molecules and indirect signaling *via* cytokines (58).

### B cells

There are limited literature about the role of B cells in the pathogenesis of DN. IgG^+^ B cells shown modestly increased in glomeruli of non-obese diabetic mice (59). After depletion B cells in these mice, the re-emerging B cells exhibit an immunosuppressive phenotype and inhibit the onset of diabetes (60). Studies have shown that CD20^+^ B cells were observed in the renal interstitium of patients with type 1 or 2 diabetes mellitus, suggesting the possibility of B cell participation in DN progression (61).

In the diabetic milieu, hyperglycemia and AGEs stimulate NF-κB signaling, which plays an important role in the development and function of B cells. It has been reported that the hyperglycemic environment might directly increase the number of both antibody- and cytokine-producing B cells, and contribute to the development of DN (59). Currently, the mechanism of B cells regulating DN is poorly understood. The role of B cells contributing to DN is most likely due to the antibodies produced by B cells. These antibodies can direct against antigens such as oxLDL and AGEs and lead to the formation of immune complexes, triggering inflammation and glomerulonephritis (59). Further studies are urgently needed to uncover the function and regulatory mechanism of B cells in DN pathogenesis.

### Mast cells

Mast cells are multipotent bone marrow-derived cells rich in growth factors and inflammatory mediators (62). Regarding the production of tryptase and chymase, mast cells were divided into MC_T_ subtype and MC_TC_ subtype in humans. MC_T_ subtype only produces tryptase, whereas MC_TC_ subtype produces both tryptase and chymase (63). In the experimental animal model of DN, there is evidence that mast cells infiltrate the kidney (64). In patients with DN, the number of mast cells increased with the progression of DN (62). Increased mast cell numbers and degranulation levels were significantly associated with tubulointerstitial injury, suggesting the mast cells are involved in development of DN (65).

Mast cells can be activated in several ways, including the well-known classical pathway, IgE-FcϵR cross-linking, and alternative pathways, such as the complement pathway and toll-like receptors pathway (65). C3a complement, the most potent activator of mast cells, has been reported to increase in DN (19). Thus, research suggests that the increased complement activation in diabetic mellitus may contribute to the recruitment and activation of mast cells. Once mass cells infiltrate into the kidney, they contribute to the pathogenesis of DN by releasing TGF-β, chymase, tryptase, renin, histamine, and inflammatory cytokines (4, 64). Specifically, mast cells may aggravate tubular interstitial fibrosis by synthesizing and releasing TGF-β and reninto initiate and promote tubular inflammation through releasing TGF-β and TNF-α (64). Further studies are needed to confirm the possible involvement of mediators by which mast cells affect the complex pathogenesis of DN.

## Immunomodulators involved in DN pathogenesis

### Soluble pro-inflammatory factors

Cytokines are a group of low-molecular-weight peptides with pharmacological activities. They have characteristic functions in autocrine and paracrine signaling, and they are important effectors of the immune system (**Table 1**).

**Table 1:** Cytokines involved in DN pathogenesis.

Cytokines	Cell Source	Cell Target	Functions	References
IL-1	Monocytes, macrophages, fibroblasts epithelial cells, endothelial cells, astrocytes	T cells, B cells, endothelial cells	Costimulatory molecule activation, acute phase reactants	(66–68)
IL-2	T cells, NK cells	T cells, B cells, monocytes	Growth and activation	(69)
IL-6	T cells, macrophages, fibroblasts	T cells, B cells	Costimulatory molecule activation, acute phase reactants	(70, 71)
IL-10	T cells	Macrophages, T cells	Inhibits APC activity and cytokine production	(72)
IL-18	Monocytes, macrophages, T cells, proximal tubular cells	T cells, NK cells	Costimulatory molecule activation, acute phase reactants	(73–75)
TNF-α	Macrophages, monocytes, T cells	T cells, B cells, endothelial cells	Costimulatory molecule activation, acute phase reactants	(68, 76–78)
TGF-β	Macrophages, T cells	Macrophages, T cells	Inhibits activation and growth	(79–81)
IFN-γ	T cells, NK cells	Monocytes, macrophages, endothelial cells	Activation increased class I and II MHC	(82)

#### ILs

IL-1 can be induced by almost all nucleated cell types, but it is mainly produced by activated macrophages and is a potent mediator of inflammation (67). In an experimental model of DN, renal IL-1 expression was found to be elevated, which was followed by expression of chemokines and adhesion molecules (66, 67). IL-1 helps drive mesangial cell proliferation and matrix synthesis, it increases vascular endothelial permeability, and it is linked to hemodynamic abnormalities within the glomerulus (83). It also upregulates ICAM-1 in certain subsets of kidney cells, such as mesangial cells, endothelial cells, and renal tubular epithelial cells (34).

Renal biopsies from DN patients show infiltration of the mesangium, stroma, and tubules by cells expressing IL-6 (67). In addition, a positive relationship was found between the severity of diabetic glomerular lesions (mesangial dilatation) and IL-6 mRNA levels in glomerular mesangial cells and podocytes, indicating that IL-6 may positively influence the dynamics of the ECM accumulation in the kidney (70). Interestingly, one study found that IL-6 regulates the differentiation of M1 macrophages into M2 macrophages through IL-4-STAT6 signaling. This finding identifies IL-6 signaling as an important determinant of macrophage activation, conferring on IL-6 an unexpected homeostatic role in limiting inflammation (71).

Among those cytokines involved in DN, IL-18 seems to be the most important one to DN pathogenesis. Elevated IL-18 levels in serum and urine have been reported in DN patients, and urinary excretion of β-2 microglobulin, a marker of tubular interstitial injury, positively correlates with serum levels of IL-18 (75, 76). Increased levels of IL-18 were found in the renal biopsies of diabetic patients in proximal tubules and epithelial cells. Serum IL-18 levels were also greater in DN patients than in healthy subjects. IL-18 is closely related to many pathogenic molecular mechanisms involved in DN. As a potent inflammatory cytokine, IL-18 promotes the production of other inflammatory cytokines, such as IL-1 and TNF-α (73). IL‐18 can also upregulate the expression of ICAM‐1, VCAM‐1, and IFN-γ in endothelial cells (74). IL-18-dependent apoptosis may play a critical role in apoptosis-induced injury in DN. Besides, IL-18 activation may lead to increased free radical production and oxidative damage (84, 85). Thus, IL-18-induced oxidative stress may be an additional mechanism by which IL-18 contributes to DN progression. Considering the vital l role of IL-18 in DN, it may become a novel therapeutic target for the prevention and therapy of DN.

#### Tumor necrosis factor alpha (TNF-α)

TNF-α, a pleiotropic inflammatory cytokine, is mainly produced by monocytes, macrophages, and T cells (66). Renal cells such as mesangial cells, glomerular cells, endothelial cells, and renal tubular cells can also secrete TNF-α in response to hyperglycemia and AGEs (86–88). The role of TNF-α in DN is supported by the detection of increased levels of the cytokine in urine from diabetic patients, and by the correlations between those levels and clinical markers of DN and disease progression (76, 77). TNF-α participates in DN progression through multiple mechanisms. TNF-α is cytotoxic to kidney cells and can induce cell apoptosis and production of ROS, as well as alter hemodynamic balance between vasoconstriction and vasodilatation (68). TNF-α increases ROS production and vice versa, which amplifies the inflammatory response (78). In rats with streptozotocin-induced diabetes, elevated TNF-α increases oxidative stress, leading to urinary albumin excretion, a marker of kidney injury (83). Other studies have shown that TNF-α significantly promotes the development of renal hypertrophy and sodium retention, both of which are characteristic alterations during early DN (34, 77, 89).

#### TGF-β

TGF-β is a major regulator of ECM production and accumulation in the diabetic kidney (90). It forwards the two milestones of DN progression, which are renal cell hypertrophy and ECM accumulation (91). Many factors of diabetic mellitus stimulate TGF-β production in the kidney. Hyperglycemia, angiotensin II, mitogen-activated protein kinase, and PKC have been shown to regulate TGF-β expression (92–95). A few studies have proven that ROS in diabetic conditions can directly or indirectly promote the production of TGF-β. Once TGF-β is activated in kidneys, it induces the production of fibronectin and collagen types I, III, and IV (79); it restrains matrix metalloproteinases, such as plasminogen activator, collagenase, elastase, and stromelysin; and it activates proteases inhibitors, such as tissue inhibitors of metalloproteinases and plasminogen activator inhibitor 1, which blocks ECM degradation (80). TGF-β positively regulates its own expression while also stimulating the deposition of ECM, thus amplifying the fibrosis response (79–81). A high glucose environment induces TGF-β expression and activation, thus pushing podocytes into the apoptosis process, which impairs filtration barrier and renal function (96). Therefore, studies targeting TGF-β signaling disruption, such as knockout of the type 2 TGF-β receptor or the downstream signaling molecular Smad3, and administration of anti-TGF-β antibodies, suspend mesangial matrix expansion and deterioration of renal function in mice (97, 98).

### Adhesion molecules

#### ICAM-1

ICAM-1 is an adhesion molecule (**Table 2**) expressed in endothelial, mesangial and epithelial cells and has been directly associated with kidney injury and DN progression in a rat model (99, 100). ICAM-1 can bind to integrins on the surface of leukocytes to promote their adhesion to endothelial cells and transmigration (68). ICAM-1 expression is upregulated in response to pro-inflammatory factors, especially TNF-α (105). Altered hemodynamic conditions resulting from TGF-β-induced ECM accumulation are also one of the factors contributing to ICAM-1 up-regulation. In addition, oxidative stress can also promote ICAM-1 expression (106). In renal mesangial and endothelial cells, AGEs induce the production of ROS, which activates NF-κB and promotes the release of pro-inflammatory cytokines and adhesion molecules (107). ICAM-1 plays a critical role in the leukocytes migration, especially T cells to the kidney (101). Deleting ICAM-1 in diabetic mice ameliorated symptoms of DN, such as glomerular hypertrophy, mesangial matrix expansion, and proteinuria (101).

**Table 2:** The type and function of adhesion molecules.

Adhesion molecules	Gene Family	Functions	References
ICAM-1	Immunoglobulin superfamily	Adhesion, rolling and crawling of leukocyte	(99–102)
ICAM-2	Immunoglobulin superfamily	Crawling of leukocyte and initiation of diapedesis	(24, 102)
VCAM-1	Immunoglobulin superfamily	Adhesion, rolling and crawling of leukocyte	(29, 103, 104)
ESAM	Immunoglobulin superfamily	Increased endothelial permeability and initiation of diapedesis	(22, 24)

#### VCAM-1

Similar to ICAM-1, VCAM-1 also involved in the leukocyte-endothelial adhesion that helps recruit leukocytes to the kidney during inflammation. In kidney interstitium of diabetic KKAy mice, VCAM-1 is upregulated on the endothelial cells of venules, and it is expressed in infiltrating cells (103). In DN patients, VCAM-1 is upregulated in kidney and as a soluble form in plasma (29). VACM-1 levels correlate with the number of infiltrating immune cells in kidney and are associated with severity and progression of albuminuria (22, 24, 104).

### Chemokines

#### MCP-1

Previous *in vivo* and *in vitro* studies have shown that differential expression of chemokines and their receptors precisely orchestrate molecular mechanisms that lead to immune cell migration in DN progression. Among them, MCP-1, also known as CC chemokine ligand 2 (CCL2), has been proposed as marker of the degree of tubular injury and renal inflammation in DN (108). In mice model of diabetes-induced renal injury, MCP-1 levels progressively increase in the kidney. Furthermore, *in vitro* studies indicate that MCP-1 expression increases in the presence of high amounts of glucose (109), and animal models of type 1 and 2 diabetes show reduced renal damage after knockout of MCP-1 (4, 15, 31). In the clinic, urinary MCP-1 levels are obviously higher in patients with microalbuminuria or albuminuria diabetes than in patients with normoalbuminuria diabetes or in healthy controls. Moreover, urinary MCP-1 levels increase as DN progresses, and they are significantly associated with other risk factors for DN (110).

Several factors were associated with the expression of MCP-1, such as hyperglycemia, TGF-β, NF-κB, PKC, ROS, and AGEs (34). There is evidence that angiotensin II also promotes MCP-1 expression. Blocking renin-angiotensin system with angiotensin converting enzyme inhibitors or angiotensin II receptor blockers significantly down-regulated the MCP-1 level in kidney cells. MCP-1 promotes the transmigration of macrophages across endothelial cells to kidney, which is the main process in the homing of macrophages in DN (111, 112). It also promotes the migration of T cells and dendritic cells to the diabetic kidney (113, 114).

### Transcription factors

Previous studies have thoroughly reviewed transcription factors involved in DN, including NF- kB, Janus kinase-signal transducer and activator of transcription (JAK-STAT), upstream stimulatory factors 1 and 2, activator protein 1, cAMP-response-element-binding protein, nuclear factor of activated T cells, and stimulating protein 1 (115). In this review, we will briefly discuss the two most vital transcription factors, NF-κB and JAK/STAT, and their roles in DN.

#### NF-κB

**Figure 3:**
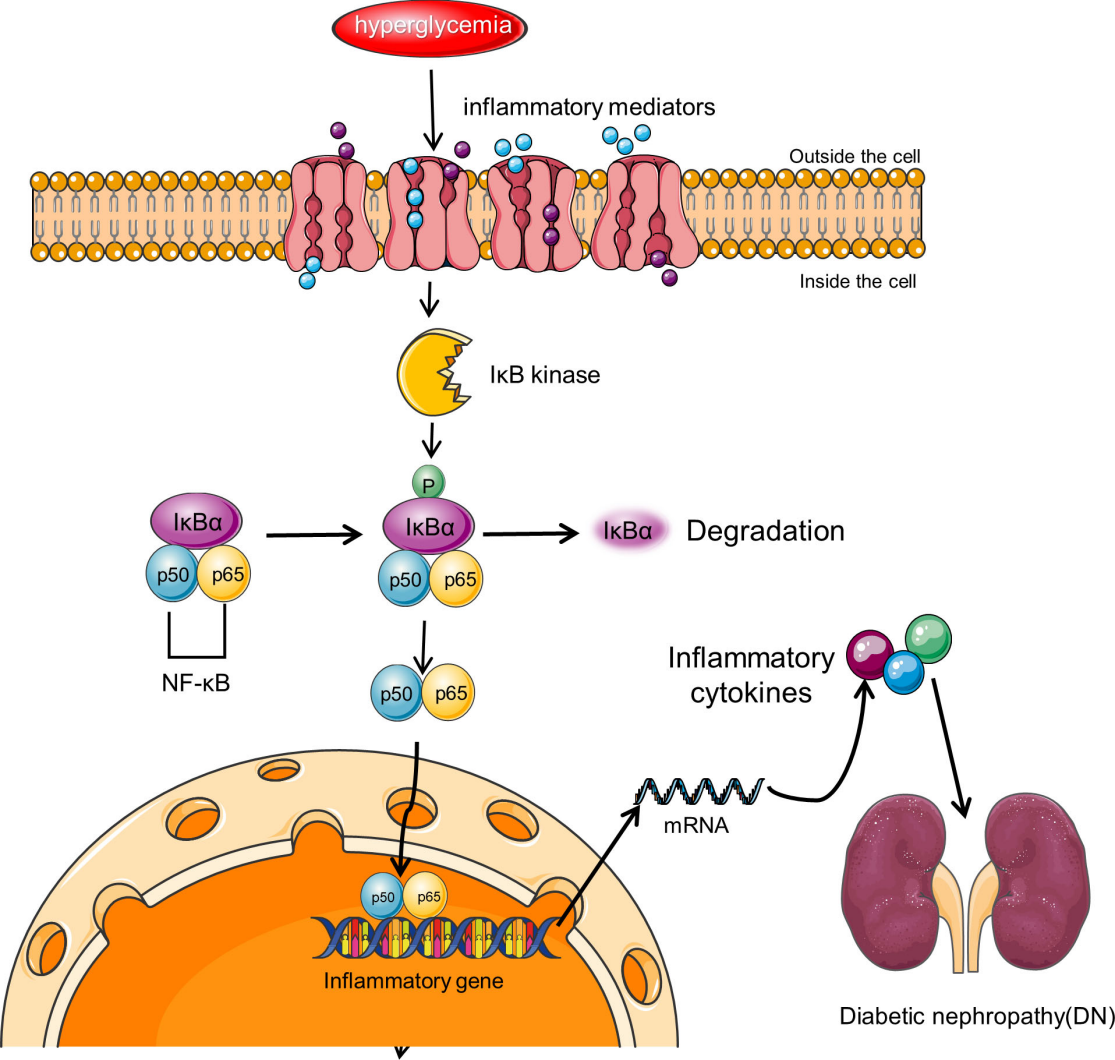
NF-κB signaling pathway in DN. NF-κB is a transcriptional regulator expressed in the cytoplasm of almost all cell types, and its activity is controlled by the IκB regulatory protein family. Activation of NF-κB involves the inhibitory protein IκB kinase being phosphorylated by specific IκB and subsequently degraded by proteolysis. Free NF-κB translocates to the nucleus, binds to promoter and enhancer sites, and activates transcription. NF-κB signaling pathway leads to increased transcription of target genes encoding inflammatory cytokines and other target genes associated with this complication, resulting in renal inflammation.

NF-κB is believed to be a master switch in the control of inflammation and is involved in the transcription of numerous genes involved in the pathogenesis of DN (**Figure 3**) (116), such as those giving rise to angiotensinogen, cytokines, and adhesion molecules (117–119). In diabetic rat models, NF-κB activation upregulates the levels of pro-inflammatory cytokines TNF-α and IL-1β (116). Upregulation of NF-κB has been indicated in monocytes of peripheral blood from patients with diabetes, and the extent of upregulation correlates with DN severity (120). Activation of NF-κB and transcription of certain pro-inflammatory chemokines in tubular epithelial cells are markers of progressive DN. Albuminuria may be one of the major pro-inflammatory phenotypes resulting from NF-κB activation (121).

#### JAK-STAT

**Figure 4:**
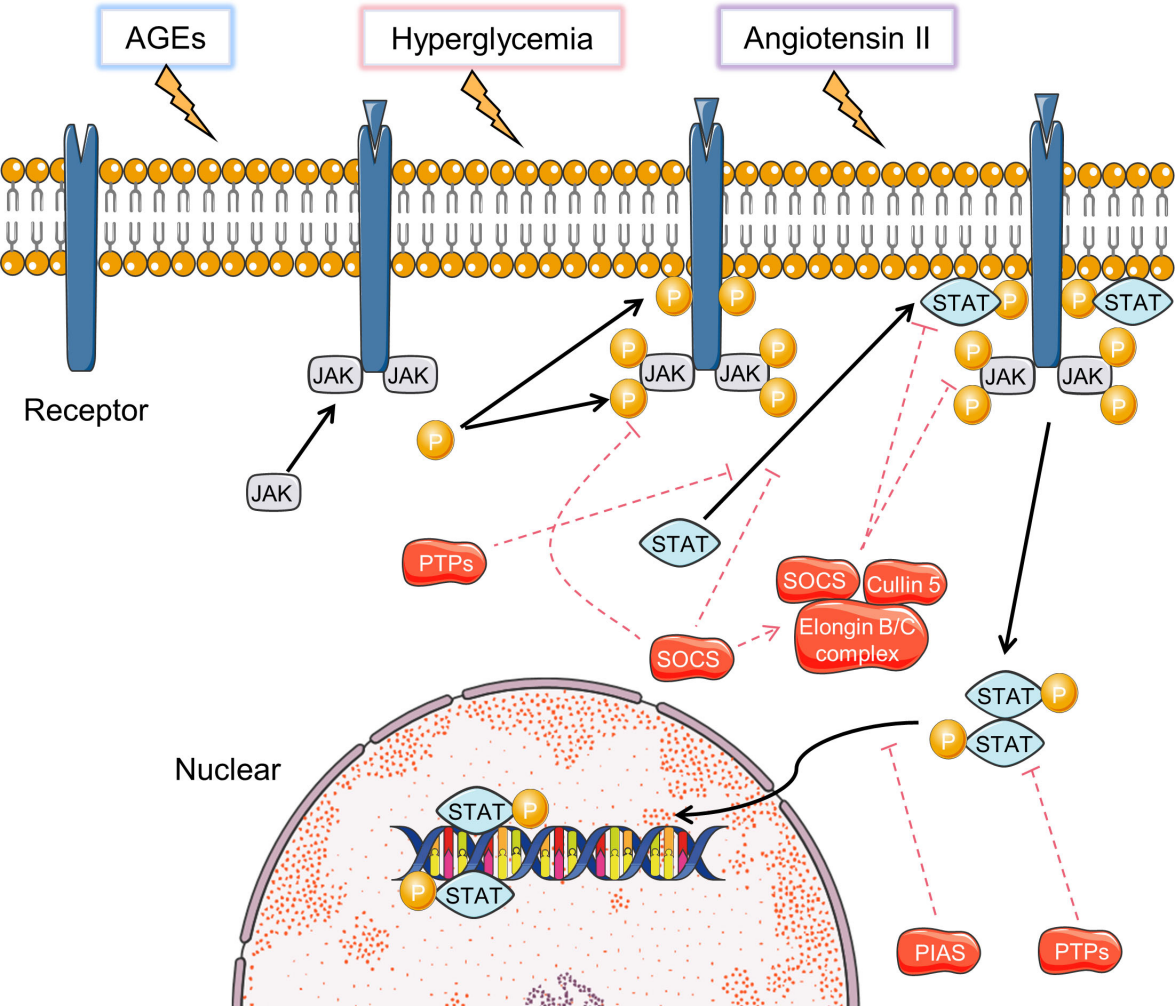
Activation and inhibition of JAK-STAT signaling pathways. Black arrows indicate the activation process and the red dotted arrows indicated inhibition process.

The JAK-STAT signaling pathway includes a family of intracellular signaling molecules that initiate activation of target genes encoding growth factors, hormones, and cytokines (**Figure 4**) (122). Studies have shown that high glucose can activate the JAK-STAT signaling in rat renal mesangial cells and in mice renal cortex at early stages of DN (123, 124). Genome-wide transcriptome analysis of DN patients showed upregulation of JAK1/2 and STAT1/3 (125). In diabetic mice, the JAK-STAT signaling is over-expressed, as is its downstream target gene encoding “suppressor of cytokine signaling (SOCS) 3”, and its upstream regulatory gene SIRT1 (126).

Hyperglycemia-induced JAK-STAT activation is a vital mechanism of renal injury in DN (127). Hyperglycemia can increase the production of angiotensin II, which in turn induces JAK2 through enhanced oxidative stress. ROS has been suggested as a mediator of hyperglycemia to regulate JAK protein activation (127). In diabetic environment, AEGs and MAPK activation can promote the acetylation and phosphorylation of STAT3 in mice and human diabetic kidneys, leading to enhanced STAT3 transcriptional activity (128–130). Transgenic mice with reduced STAT3 activation ability are protected from inflammation and injury in the diabetic kidney (131). Overexpression of SOCS-1 and SOCS-3, which are negative regulators of JAK-STAT signaling, reduce macrophage infiltrations, levels of pro-inflammatory cytokines, renal injury in rodents with DN (126). The current researches mainly focus on JAK1/2 and STAT3. Therefore, future studies on the role of other JAKs and STATs may aid in revealing novel regulatory mechanisms of DN.

### Other immune processes

#### Complement system

The complement system is an essential part of the innate immune systems, which can enhance the ability of antibodies and phagocytes to clear microbes and damaged cells (8). The complement system also promotes inflammation (23). Growing evidence has shown that complement system is involved in the progression of DN (19). According to transcriptome and immunohistochemical analysis of renal biopsies, 50-60% of DN patients have glomerular deposition of complement component C3, and such deposition is associated with severity of glomerulosclerosis (125). The glomerular deposition of complement C3 is also a characteristic of DN animal models associated with type 1 or 2 diabetes, and such deposition has been linked to glomerular deposition of immunoglobulin G (IgG), which induces inflammation and damages the kidney tissue by producing chemokines (4, 132, 133).

#### Nucleotide-binding oligomerization domain-like receptor pyrin domain containing 3 (NLRP3) inflammasome

The inflammasome assembles during DN immune responses in a way that drives the pathology of kidney diseases. NLRP3 is by far the best characterized inflammasome in the kidney (21, 134). The activation of NLRP3 inflammasome in immune cells generally requires two steps: priming and activation. The priming step is stimulated by the binding of pathogen-associated molecular patterns (PAMPs) and/or DAMPs to toll-like receptors and/or cytokine receptors. This step often involves the activation of NF-κB signaling and regulation of downstream genes that increase the expression of inflammasome-associated genes and substrates (10, 21). Following priming, the activation step involves NLRP3 oligomerization and the assembly of inflammasome components into a complex. By cleaving pro-caspase-1 into caspase-1, the resulting complex promotes the maturation and secretion of IL-1β and IL-18, further leading to the accumulation of mesangial cells, podocyte damage, and albuminuria (10, 134).

The expression of NLRP3 is elevated in the glomerulus of mouse DN models. Thus, NLRP3-knockout animal models are necessary to reveal the vital role of NLRP3 inflammasome in DN. Indeed, one study demonstrated that level of IL-1β in podocytes was significantly upregulated in STZ-induced diabetic mice, which was reversed in NLRP3 knockout mice (135). Deleting NLRP3 significantly prevented the accumulation of glomerular neutral lipid and cholesterol in diabetic mice (136).

These studies make clear that the immune system plays an essential role in the progression of DN. Below, we review promising therapeutic targets in DN as well as therapeutic agents already under development.

## Clinical and pre-clinical therapies targeting the immune system for treatment of DN

### Inhibition of soluble pro-inflammatory mediators

#### TNF-α

Among the inflammatory mediators associated with DN, TNF-α has perhaps been best studied for its therapeutic potential: several studies have examined how its inhibition can slow DN progression (137). Infliximab is a chimeric immunoglobulin G1κ murine/human monoclonal antibody developed as a therapeutic agent against rheumatoid arthritis and Crohn’s disease (138, 139). Infliximab reduced the expression of TNF-α and improved DN symptoms in diabetic mice (140). The TNF-α inhibitor SKF86002 markedly decreased glomerulus TNF-α level and improved kidney function in patients with DN (141). Pentoxifylline (PTX), originally created to treat intermittent claudication caused by peripheral vascular diseases (142–144), has shown potential for mitigating proteinuria and restoring glomerular filtration in the context of diabetic kidney disease. PTX inhibits TNF-α expression as well as the activity of other inflammatory mediators, such as IL-1, IL-6, IFN-γ, VCAM-1 and ICAM-1 (145–147). Future studies are needed to clarify whether PTX can improve renal outcomes in DN.

#### TGF-β

Direct inhibitors of TGF-β can efficiently block the progression of DN (148). But indirect inhibition has also shown benefit (149, 150). Melatonin, a hormone secreted by the pineal gland, may improve kidney inflammation and interstitial fibrosis in DN by inhibiting the TLR4 and TGF-β/Smad3 signaling pathways (150). Given that melatonin is also capable of reducing urinary excretion and protecting podocytes (151), it may prove a promising therapeutic in DN. Sitagliptin is a dipeptidyl peptidase-4 (DPP-4) inhibitor best known for its hypoglycemic properties (152). In diabetic mice, sitagliptin improved renal function by inhibiting the TGF-β/Smad signaling pathway (153). Dencichine is a non-protein amino acid, originally extracted from *Panax notoginseng* (154), that may treat DN by reducing hyperglycemia, restoring metabolic disorder, reducing ECM deposition, increasing the activity of enzymes that degrade the ECM, and down-regulating TGF-β/Smad signalling in DN glomeruli (155).

#### MCP-1

Breviscapine and triptolide act as MCP-1 receptor antagonists in animal models of DN, reducing downstream signaling pathways that induce ROS production and inflammation (156). Breviscapine, extracted from the Chinese herb *Erigeron breviscapus*, may indirectly mitigate DN by reducing albuminuria (156). In contrast, triptolide regulates the proportion of Th1/Th2 cells, reduces MCP-1 expression, and inhibits macrophage infiltration as well as expression of related inflammatory factors in the kidney (157–160). Other inhibitors, such as the CCR2 inhibitor CCX140-B and the MCP-1/CCL2 inhibitor NOX-E36, are currently in pre-clinical studies or clinical trials (161–164). In a murine model of DN, NOX-E36 significantly reduced glomerulosclerosis and improved glomerular filtration rate (163), while CCX140-B significantly reduced proteinuria in DN patients (161).

### Inhibition of transcription factors

#### NF-κB signaling inhibitors

Inhibitors of NF-κB have been used to mitigate DN and inflammatory injury of the kidney, as well as improve kidney function (165). Thiazolidinediones, agonists of peroxisome proliferator-activated receptor (PPAR)-γ, are widely used as insulin sensitizer in diabetes therapy (166, 167). These ligands repress renal injury in an experimental rat DN model by inhibiting NF-κB activity (168). Cultured renal tubular epithelial cells pretreatment with15a, a derivative of salviadione, prevented high glucose induced NF-κB activation and expression of inflammatory cytokines (169). In mice with streptozotocin-induced diabetes, the antioxidant tocotrienol suppressed NF-κB activation, reduced TNF-α and TGF-β levels and reversed renal dysfunction (4, 170). Treating these animal model with BAY-110782, an inhibitor of IκB, or pyrrolidine dithiocarbamate, an inhibitor of NF-κB, reduced NF-κB activation, renal macrophage infiltration and production of the inflammatory cytokines MCP-1, TNF-α, IL-1β and IL-6 (171, 172).

#### JAK-STAT signaling inhibitors

Various drugs and compounds may show anti-inflammatory effects in DN by inhibiting JAK-STAT signaling (131). Paeoniflorin, a monoterpene glycoside extracted from the dried root of *P.lactiflora* Pall, downregulates the phosphorylation of JAK2 and STAT3 in diabetic kidney (173). Baricitinib, a selective inhibitor of JAK1 and JAK2, reduced albuminuria in patients with DN associated with type 2 diabetes in phase 2 randomized clinical trials (174, 175). Others inhibitors of various JAK proteins, such as ruxolitinib and tofacitinib, have already been approved for clinical use by the US Food and Drug Administration (175, 176).

### Inhibition of other immune processes

#### Inhibitors of the complement system

To date, only a few studies have reported the efficacy of blocking complement system in DN (19, 23). The lectin-like domain of thrombomodulin constrained glucose-induced complement activation on podocytes an endothelial cells and ameliorated albuminuria and glomerular damage in mice (177). Treatment with receptors of the complement fragments C3a/C5a may ameliorate DN by partially blocking the endothelial-myofibroblast transition and fibrosis through inhibition of the Wnt/β-catenin signaling pathway (178). Similarly, in a diabetic rat model, administration of C3a receptor improved DN pathogenesis by inhibiting IκBα phosphorylation and TGF-β/Smad3 signaling, which reduced the cytokine release and ECM accumulation (179).

Abnormal regulation of the complement cascade leads to immune and non-immune types of kidney damage (19). This insight into the pathological mechanisms related to complement and regulators will aid the development of new therapies. Monoclonal humanized antibody eculizumab, that binds C5 and prevents assembly of the membrane attack complex (C5b-9), is already in clinical use (180). Complement-targeting therapy is expected to exert a more important role in the treatment of DN in the future.

#### NLRP3 inflammasome inhibitors

MCC950, a small molecule inhibitor of NLRP3, can specifically and potently inhibits NLRP3 inflammasome activation (181). MCC950 is reported to improve podocyte injury in DN by inhibiting lipid accumulation, ROS production and p65 activation (135). CY-09 is another NLRP3-specific inhibitor, and it blocks oligomerization of the NLRP3 inflammasome (182). Furthermore, it downregulates blood glucose and insulin level, improves glucose tolerance and decreases hepatic steatosis in diabetic mice, suggesting that it may exert therapeutic effects against type 2 diabetes. In fact, CY-09 reduces the levels of IL-1β in the serum, liver and adipose tissue of diabetic mice, without affecting metabolic parameters in control mice (183). Oridonin is a the main ingredient of the traditional Chinese herb *R.rubescens* that significantly attenuates diabetes-induced renal injury by dampening inflammatory responses, based on studies *in vitro* and *in vivo* (183). Oridonin appears to prevent NF-κB from binding DNA and turning genes on (184). Tranilast is a cell membrane stabilizer that has been widely used in the treatment of inflammatory diseases because it inhibits the release of histamine and other chemical mediators (185). Tranilast prevents NLRP3 assembly by inhibiting interactions of NLRP3 with other NLRP3 molecules or with apoptosis-associated speck-like protein containing a C-terminal caspase activation and recruitment domain. Tranilast blocks the ability of a high fat diet to upregulate IL-1β in the serum, liver, or adipose tissues of diabetic mice. Tranilast also suppresses caspase-1 cleavage in diabetic mice, suggesting that the drug can inhibit metabolic stress-induced inflammasome activation (186).

### Hyperglycemia therapies that dampen immune responses

#### Sodium‐glucose cotransporter‐2 (SGLT2) inhibitors

SGLT2 inhibitors, which alleviate hyperglycemia by stimulating the excretion of glucose into urine, have been approved for the treatment of type 2 diabetes (187). Since persistent hyperglycemia is a central cause of DN progression, SGLT2 may also be effective against that renal complication (188). SGLT2 blocks glucose reabsorption at the proximal tubule, leading to glucosuria and lowering of blood glucose levels, which is independent of insulin (189). Treating diabetic animals with empagliflozin or ipragliflozin reduces their hyperglycemia and reduces levels of pro-inflammatory cytokines and chemokines, NF-κB and C-reactive protein in kidney or plasma (190–192). Dagagliazine mitigates hyperglycemia and diabetic tubulointerstitial injury by suppressing inflammatory markers and oxidative stress in the renal tissues of diabetic mice (193). Similarly, dapagliflozin blocks oxidative stress, inflammation and apoptosis induced by high glucose, and it promotes renal function and angiogenesis by upregulating vascular endothelial growth factor (194). Canagliflozin decreases plasma levels of IL-6, matrix metalloproteinase-7, TNF receptor 1, and fibronectin 1 in human, suggesting that it may mitigate inflammation, ECM deposition and fibrosis in DN (195).

### Promising novel therapy directions

#### MicroRNAs

MicroRNAs are important mediators of the post-transcriptional feedback control mechanism and participate in metabolism and inflammation regulation. Pioneering work with microRNAs has provided a new outlook on molecules and signaling pathways involved in DN pathogenesis (**Table 3**). MicroRNAs are non-coding RNAs that regulate gene expression through epigenetic mechanisms and may therefore allow design of drugs that could prevent DN before it appears (222–224). Both miR-192 and miR-21 have been implicated in renal fibrosis, albeit through different mechanisms (196, 225). Of note, miR-192 is involved in a negative feedback loop with TGF-β signaling (226). Thus, these miRNAs deserve further investigation as targets in the treatment of DN. Indeed, knockdown of miR-21 in the kidneys of diabetic *db/db* mice improved renal function and inhibited renal fibrosis and inflammation during DN associated with type 2 diabetes (197). Induction of renal protective miRNAs and silencing of injury-induced miRNAs in patients with DN have been shown to restore renal function (222). Currently, several miRNAs-based preparations have entered clinical trials, such as Miravirsen, an inhibitor of miR-122 for hepatitis C treatment (227), and MRX24, a liposome-based miR-34 mimic for the treatment of cancer (228). We believe that microRNAs-based preparations may also apply to the treatment of DN in the future.

**Table 3:** The miRNAs involved in regulating the immune mechanism of DN.

MicroRNAs	Expression in DN	Targets	Functions	References
miR-21	Up-regulated	MMP9/TIMP1, Smad7, PPAR-α	Increasing fibrosis and inflammation	(196–198)
miR-23a	Up-regulated	Ubiquitin editor A20	Macrophage activation and renal tubulointerstitial inflammation	(199)
miR-20b	Down-regulated	Kruppel-like family gene, TXNIP, IL-8	Increasing renal inflammatory response	(200)
miR-19b-3p	Up-regulated	SOCS-1 gene	M1 macrophage activation and renal tubulointerstitial inflammation	(201)
miR-29b	Down-regulated	Sp1 gene and T-bet gene	Increasing microalbuminuria, renal fibrosis, and inflammation	(202)
miR-29c	Up-regulated	Sprouty homolog 1	inducing apoptosis and increasing fibronectin synthesis in podocytes	(203)
miR-27a	Up-regulated	Nrf2/Keap1 pathway	Increasing Inflammation and oxidative stress	(204)
miR-31	Down-regulated	E-selectin	Increasing inflammation and interaction between leukocytes and endothelial cells	(205)
miR-124	Up-regulated	Integrin α3	Damaging podocytic adhesive capacity	(206)
miR-93	Down-regulated	Vascular endothelial growth factor A	Increasing microalbuminuria and leading to thrombotic glomerular injury	(207)
miR-192	Up-regulated	E-box repressors(δEF1 and SIP1)	Increaseing renal fibrosis and proteinuria	(208, 209)
miR-195	Up-regulated	SIRT1	Reducing the apoptosis of renal mesangial cells	(210, 211)
miR-200a	Down-regulated	TGF-β2	Reducing Renal Fibrogenesis	(212)
miR-802	Up-regulated	NF‐κB‐repressing factor	NF‐κB activation and renal inflammatory response	(213)
miR-455-3p	Down-regulated	Rho-associated coiled coil-containing protein kinase 2	Reducing glomerular hypertrophy, mesangial amplification, and renal fibrosis	(214)
miR-374a	Down-regulated	MCP-1	Reducing renal inflammatory response	(215)
miR-544	Down-regulated	Fatty acid synthase	Reducing glomerulosclerosis and renal inflammation	(216)
miR-346	Down-regulated	Smad3/4	Reducing renal fibrosis	(217)
miR-451	Down-regulated	LMP7, PSMD11, NF‐κB	Promoting the expression of pro-inflammatory molecules and proliferation of mesangial cells, resulting in glomerular injury	(218, 219)
miR-199a-3p	Down-regulated	Inhibitor kappa B kinase β	Reducing high glucose−induced apoptosis and inflammation	(220)
miR-377	Up-regulated	PAK1, SOD1/2	Increasing fibronectin production and inflammation	(221)

#### Stem cells and stem cells-derived exosomes

Stem cells are a class of cells that have the ability to renew themselves indefinitely and differentiate into multiple cell lineages (229). Stem cells can be classified according to their differentiation capability: (1) pluripotent stem cells; (2) multipotent stem cells; (3) unipotent stem cells (230). Mesenchymal stem cells (MSCs) are one of the most widely studied pluripotent stem cells in DN (231). Among these stem cells, MSCs have several advantages to apply in DN therapy, such as easy harvesting, multi-lineage differentiation potential, strong immunosuppression, and no immune rejection (232). MSCs come from a wide range of sources, including bone marrow, adipose tissue, umbilical cord blood, peripheral blood, and amniotic fluid, among which bone marrow is the most abundant source (233–237). MSCs can differentiate into glomerular mesangial cells, tubular epithelial cells, endothelial cells, and podocytes (238–240). In STZ-induced rat DN model, MSCs injection can upregulate anti-inflammatory factors such as IL-10 and EGF, downregulate pro-inflammatory factors, and inhibit macrophage activation (241). In addition, administration of MSCs reduced pathological damage, collagen deposition, and fibrosis in the kidney (242). Although the safety and efficacy of MSCs therapy have been evaluated in clinical trials for kidney transplantation, liver fibrosis, and Crohn’s disease, the clinical trials of MSCs in DN are still ongoing (229, 243–245).

Recently, microvesicles secreted by MSCs, known as exosomes, have been widely studied in animal experiments and have demonstrated their roles in DN therapy (246–248). Exosomes containing functional proteins and RNA (microRNA and mRNA) can be detected in the MSCs medium supernatant, which contributes to cell-to-cell communication in paracrine manners (248). Therefore, many studies have focused on the role of exosomes as a key factor in the paracrine action of MSCs in DN (246, 247, 249, 250). Exosomes isolated from MSCs conditioned medium by ultrafiltration-combined purification method were administrated to STZ-induced DN rat model. The result showed significantly reduced mTOR pathway expression and fibrosis markers in renal tissue (249). Intravenously administration of MSC-conditioned medium to high-fat diet (HFD) and STZ-induced diabetic mice showed decreased proteinuria and proinflammatory cytokines expression, and significantly ameliorated tubulointerstitial fibrosis (247). Research in the coming years will focus on this secretion as a possible treatment option without significant side effects. Future studies are needed to clarify the molecular mechanism of mesenchymal-derived exosomes in improving DN.

#### Nanomedicines

Due to impaired glomerular filtration and tubular secretion function in DN, drugs can hardly reach the injured kidneys efficiently. Therefore, the treatment of kidney diseases requires high doses of the drug, which are usually associated with serious adverse effects. In recent years, the application of nanomedicines is gradually emerging in the treatment of renal diseases. Owing to the superior targetability and improved pharmacokinetic properties of nanomedicine, kidney-targeted nanomedicine carrying drug candidates can help to address the challenges associated with DN pharmacotherapy (251).

Numerous nanomedicine-based drug delivery systems have been developed to deliver therapeutic agents specifically to the kidney (252). For example, drug nanocomplexes containing low-molecular-weight chitosan bind the megalin-cubilin receptor in proximal tubules (251). Albumin nanoparticles with specific size target mesangial cells. Wu et al. reported that albumin-methylprednisolone nanoconjugates with a size of about 10 nm can specifically target the podocytes (253). These nanoconjugates avoid the side effects of glucocorticoids in patients with DN. Another investigation developed a nanoconjugate of baicalin and lysozyme with good renal targetability. This conjugate successfully ameliorated renal fibrosis and inflammation *via* NF-κB, TGF-β1/Smad3, and IGF-1/p38 MAPK signaling pathways. Manna et al. developed and studied the effect of pomegranate peel extract-stabilized gold nanoparticles (PPE-AuNPs) on the STZ-induced DN mice model (254). In DN mice, PPE-AuNPs significantly improved renal fibrosis and glomerular sclerosis. Specifically, it alleviated renal inflammation by modulating the MAPK/NF-kB/STAT3/cytokine axis.

As evidenced by the large number of nanoparticle formulations already on the market and many more in clinical trials, nanomedicines will surely take a large market share soon. Novel strategies to develop nanomedicine-based platforms with superior efficacy and safety for DN-targeted drug delivery hold great promising for the treatment of DN in the future.

## Conclusion

The global burden of diabetes seems certain to increase dramatically in the future, coinciding with the rise in obesity. This implies a corresponding increase in the incidence of DN. Despite the efficacy of hypoglycemic drugs, they will be insufficient to halt disease onset and progression as the number of new cases. Therefore, new strategies and targets against DN are urgently needed. Emerging knowledge about immune responses and inflammation as bridges in the pathogenesis between abnormal metabolism and DN offers new promising for targeted therapies. Already under investigation are therapies focusing on the regulation of inflammatory pathways and, involving targets such as immune cells, pro-inflammatory cytokines, adhesion molecules, chemokines, JAK-STAT signaling, or NF-κB signaling. Additional promising targets may be the complement system, microRNAs and downstream targets of specific inflammatory signaling pathways. It is clear that the role of the immune response in DN pathogenesis is quite complex and multi-faceted, which highlights the need to explore combination therapies.

## Author contributions

JC and YL wrote the manuscript. QL and JH contributed to the discussion and reviewed the manuscript. JH and YL obtained funding. JC, JH and YL are the guarantors of this work and as such, had full access to all the data in the study and take responsibility for the integrity of the data and the accuracy of the data analysis.

## Funding

This study was supported by the National Natural Science Foundation of China (82025007, 82170874, 81930020, and 81870599).

## Conflict of interest

The authors declare that the research was conducted in the absence of any commercial or financial relationships that could be construed as a potential conflict of interest.

## Publisher’s note

All claims expressed in this article are solely those of the authors and do not necessarily represent those of their affiliated organizations, or those of the publisher, the editors and the reviewers. Any product that may be evaluated in this article, or claim that may be made by its manufacturer, is not guaranteed or endorsed by the publisher.
